# Supplementary figures and images for: PML mutants from arsenic-resistant patients reveal SUMO1-TOPORS and SUMO2/3-RNF4 degradation pathways
Source: J Cell Biol. 2025 Apr 16;224(6):e202407133. doi: 10.1083/jcb.202407133 (PMC12002637; doi:10.1083/jcb.202407133)

YFP-  
PML-V  
WT

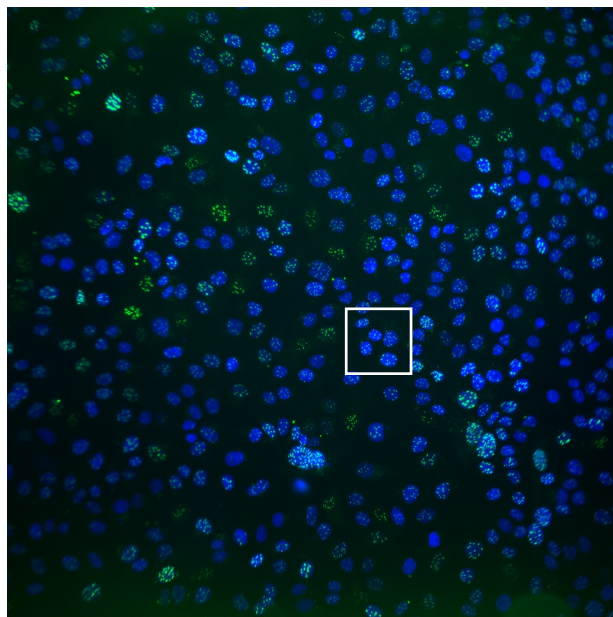

YFP-  
PML-V  
A216T

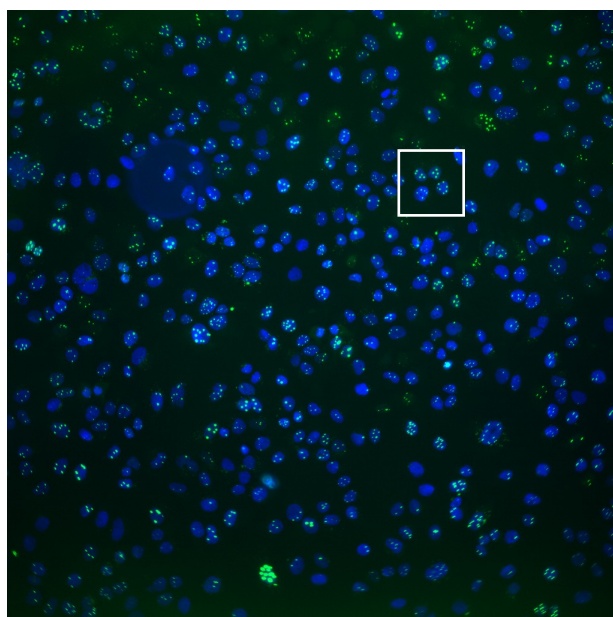

YFP-  
PML-V  
L217F

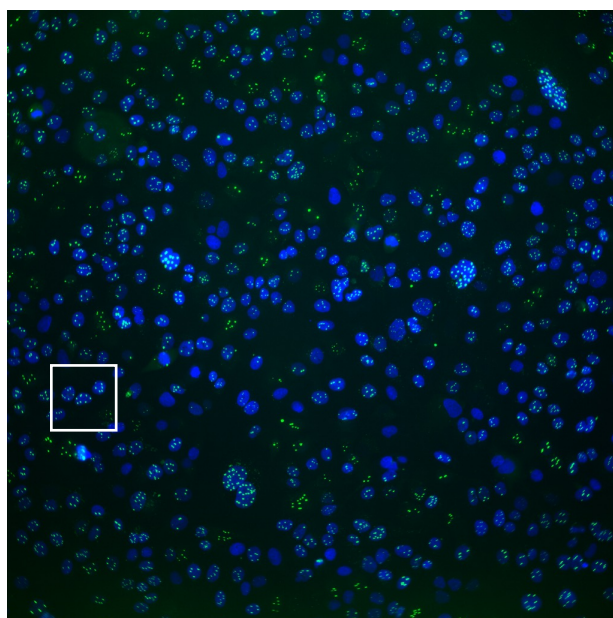

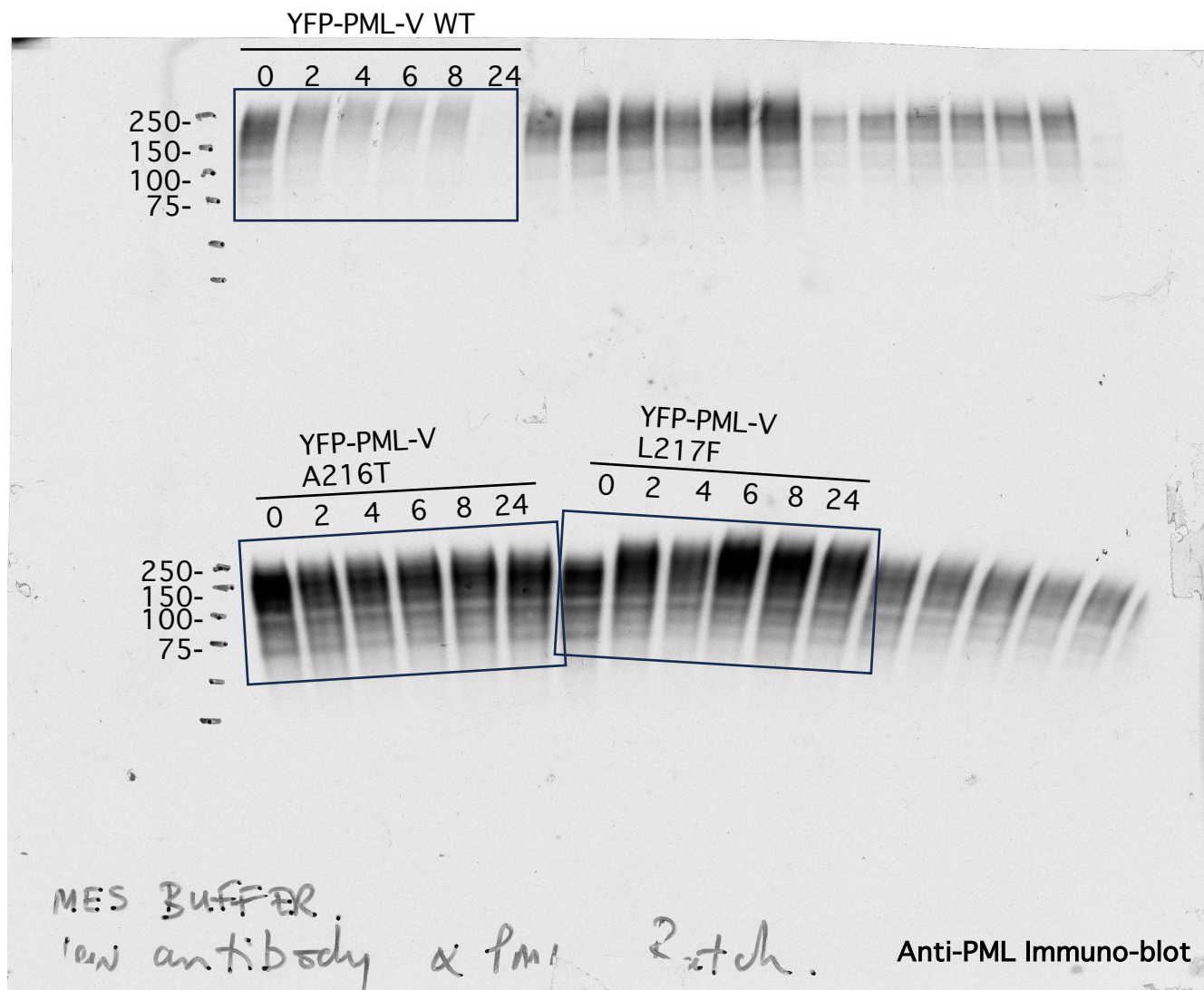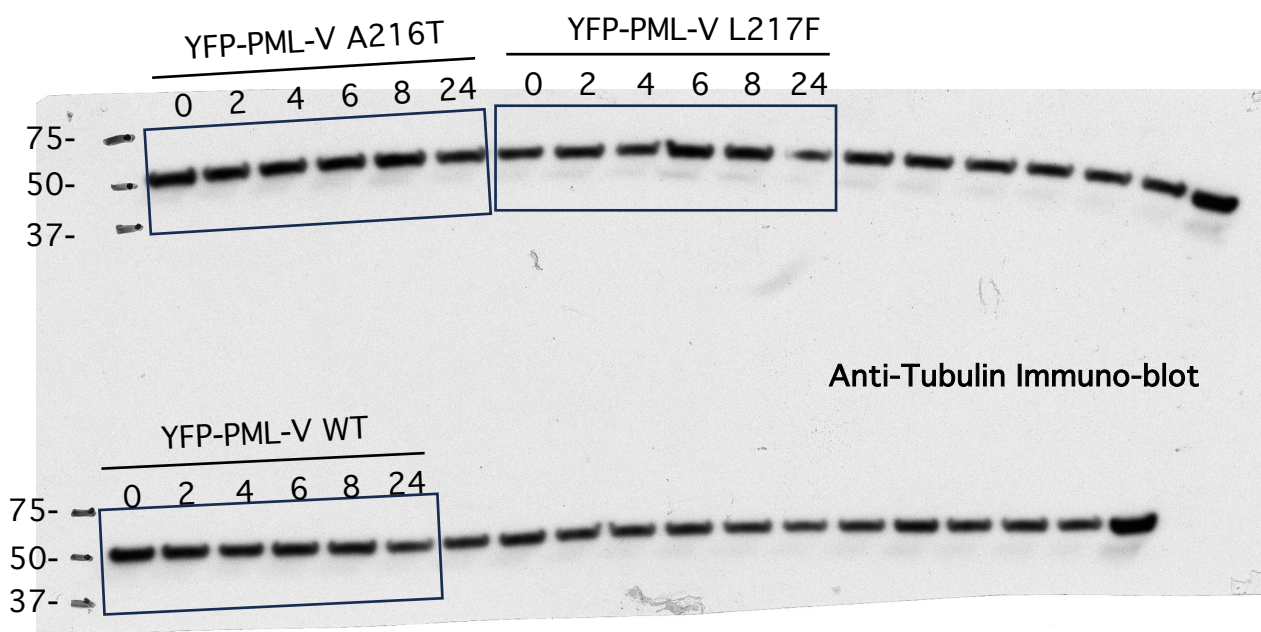

Supplement: SourceData F1 — is the source file for Fig. 1. [file jcb_202407133_sourcedataf1.pdf]

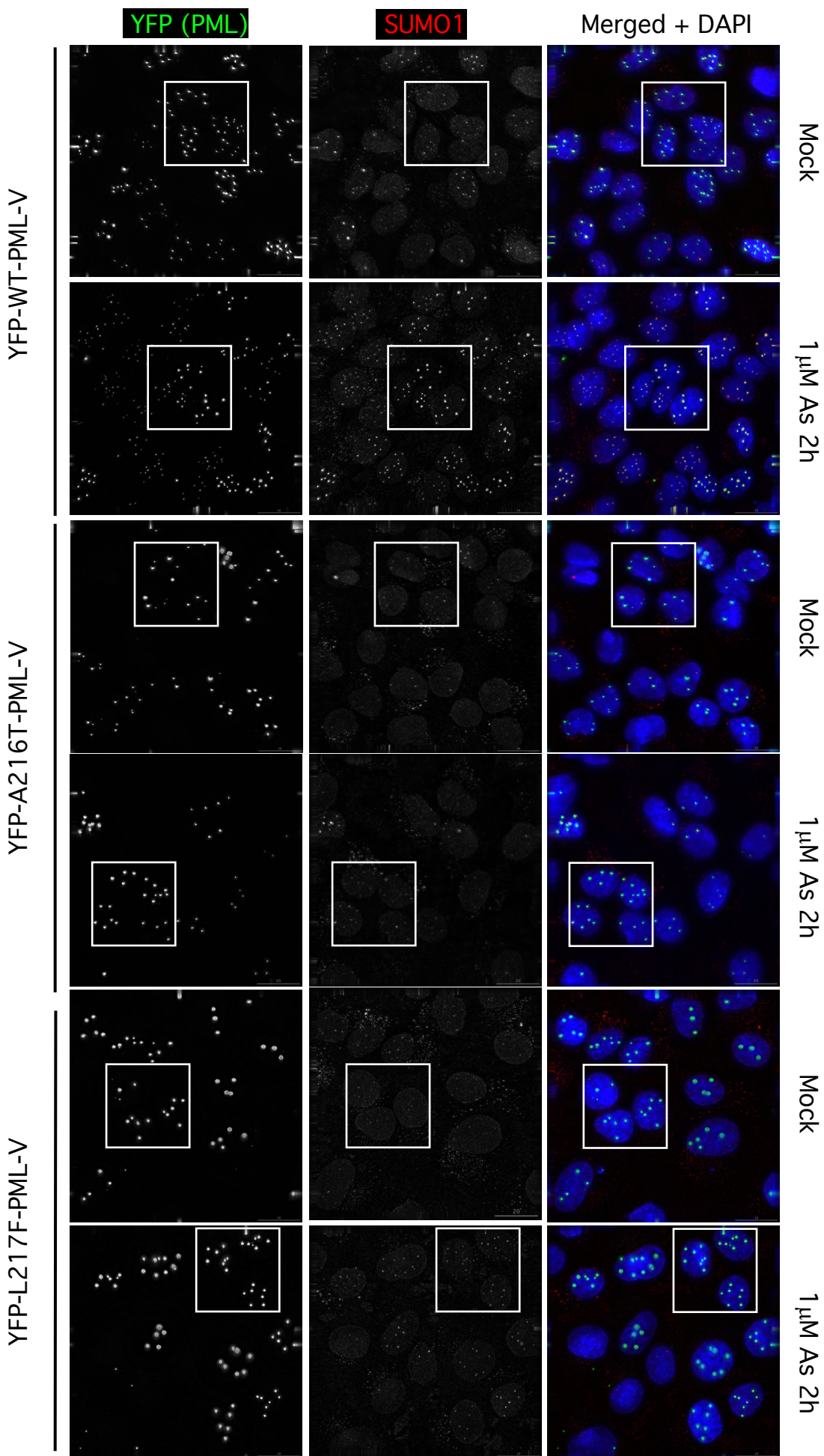

YFP (PML)

SUMO2

Merged + DAPI

YFP-WT-PML-V

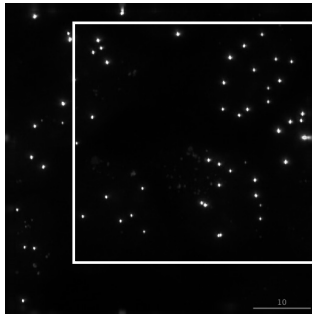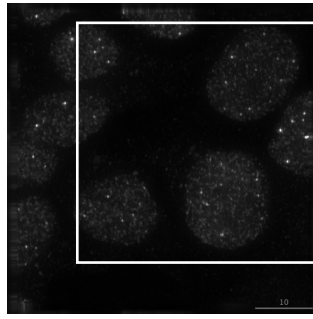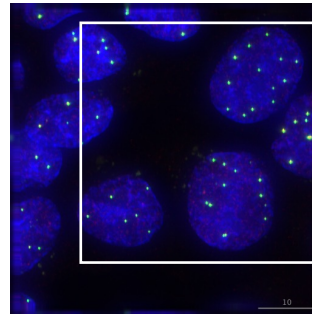

Mock

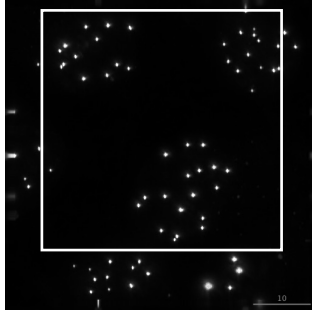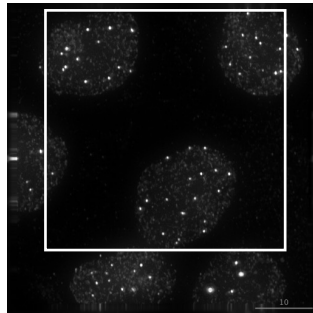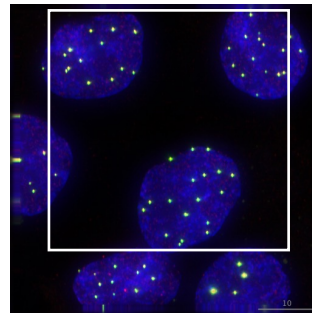

1  $\mu$ M As 2h

YFP-A216T-PML-V

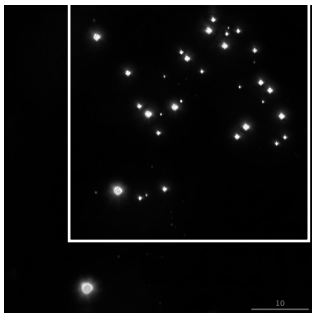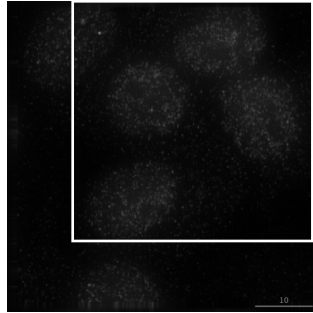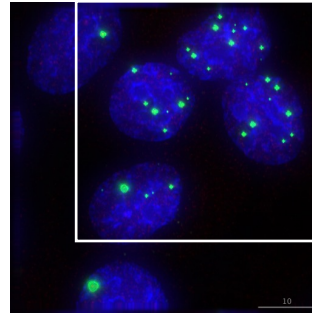

Mock

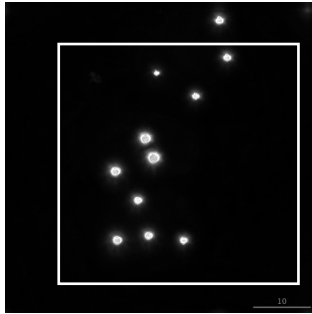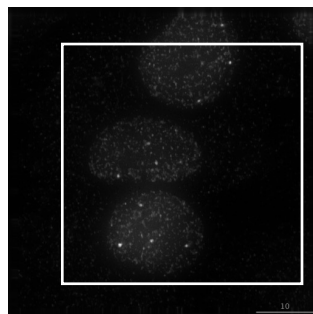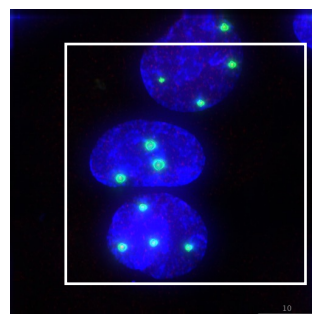

1  $\mu$ M As 2h

YFP-L217F-PML-V

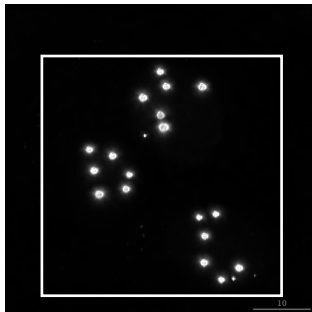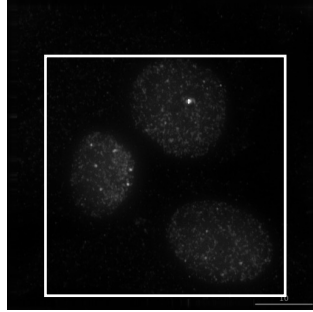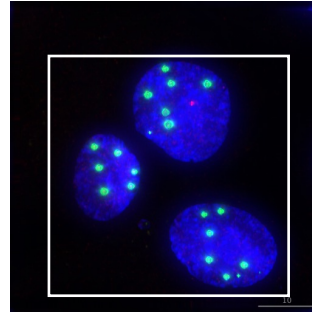

Mock

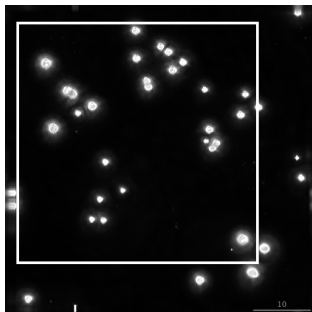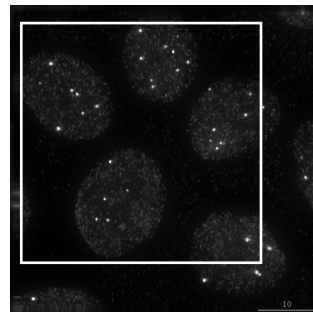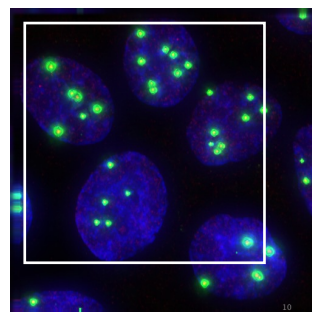

1  $\mu$ M As 2h

Supplement: SourceData F3 — is the source file for Fig. 3. [file jcb_202407133_sourcedataf3.pdf]

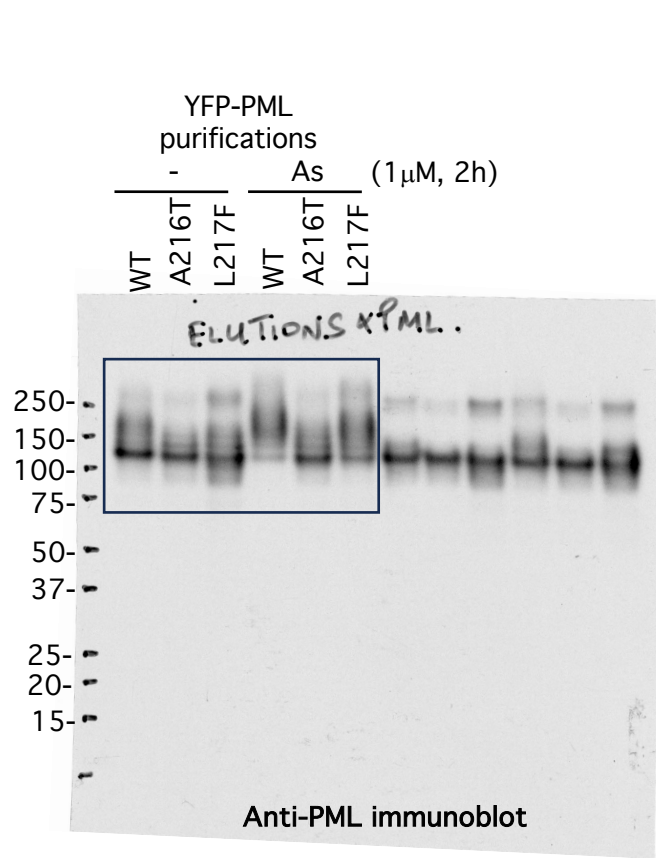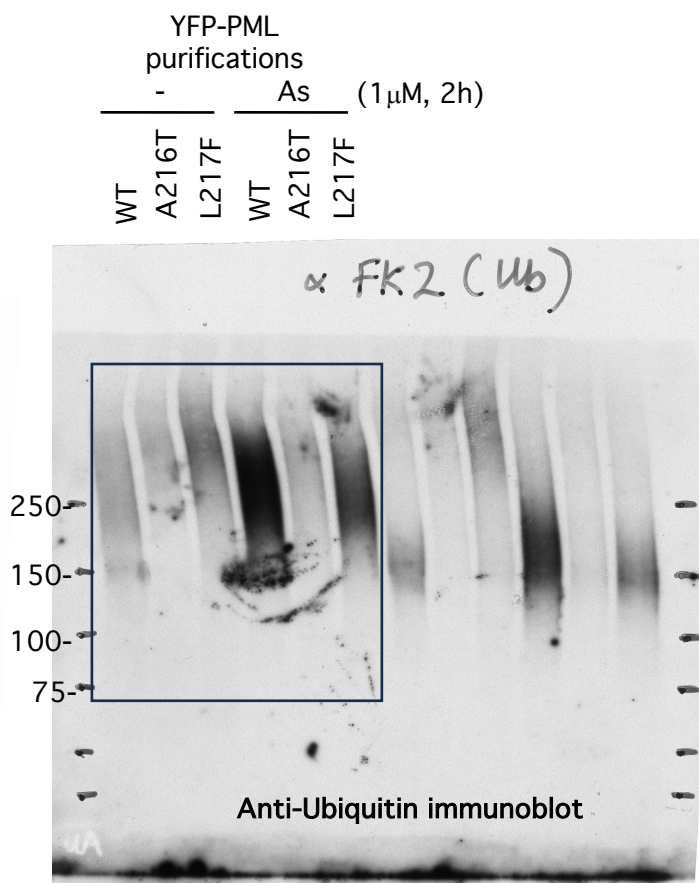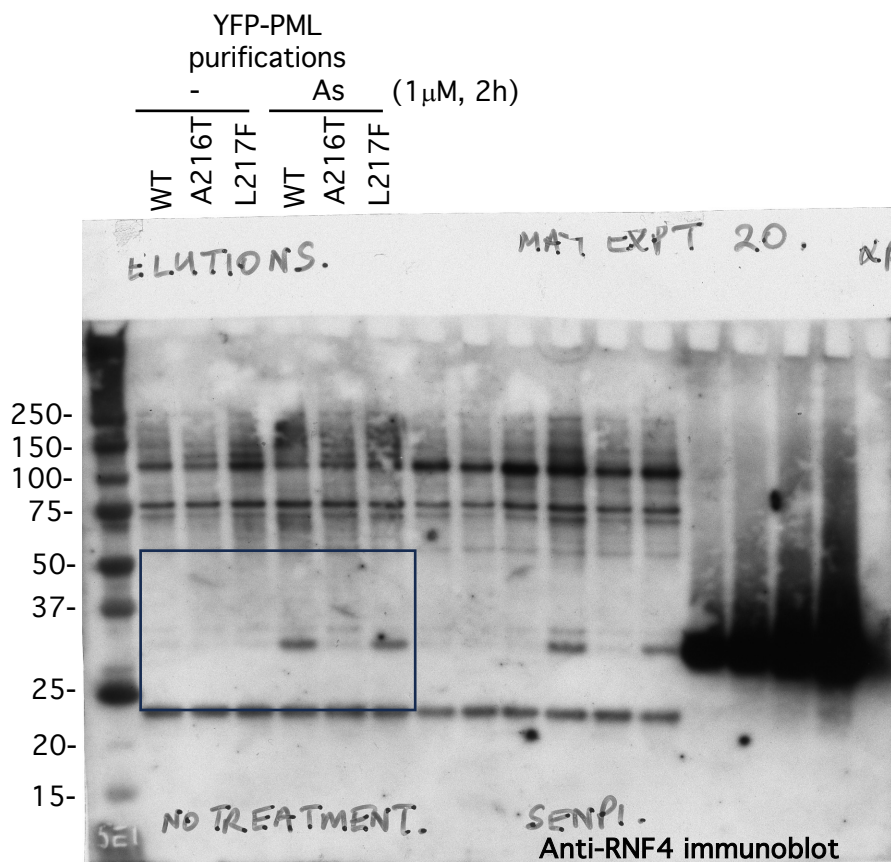

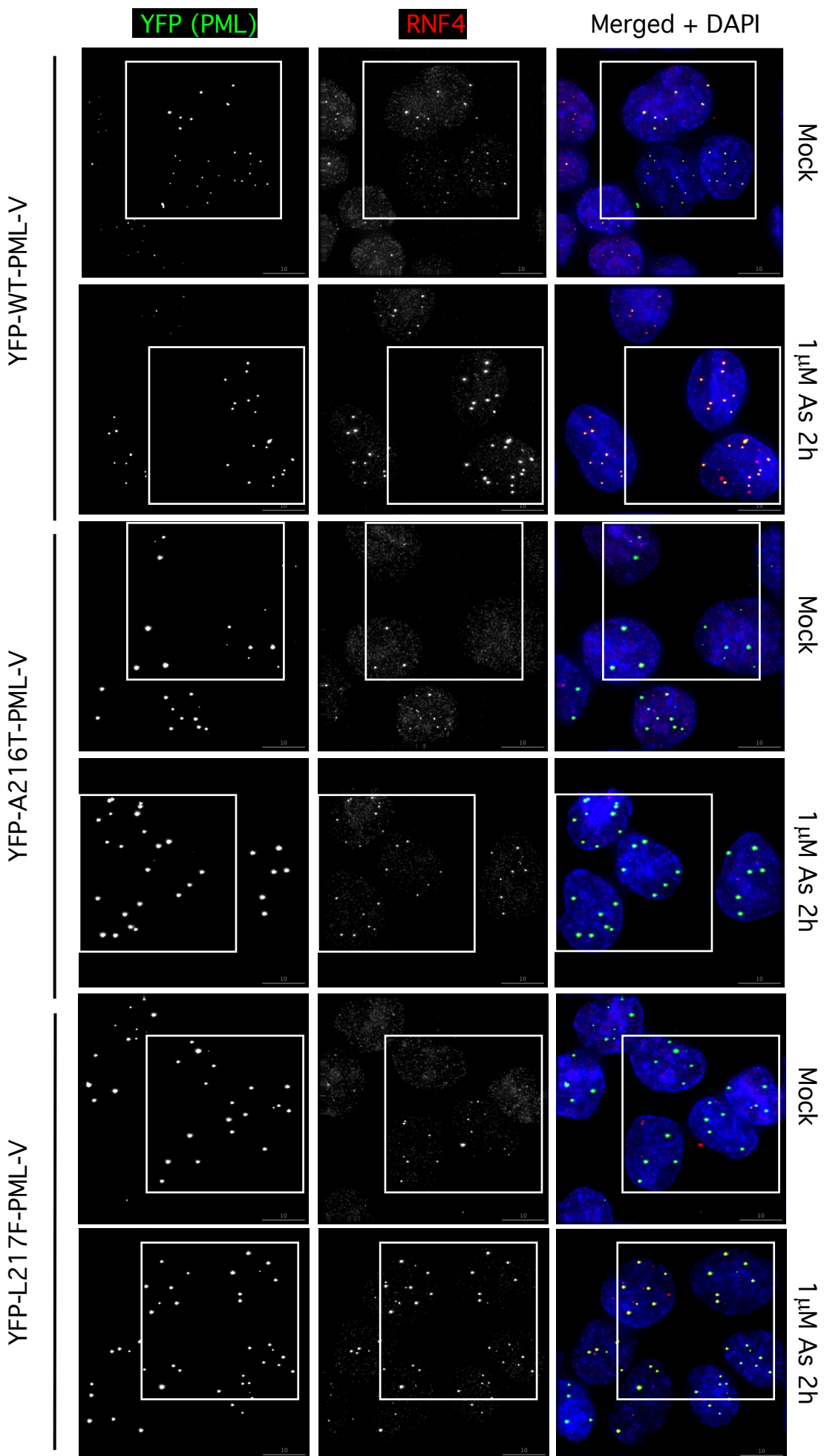

Supplement: SourceData F4 — is the source file for Fig. 4. [file jcb_202407133_sourcedataf4.pdf]

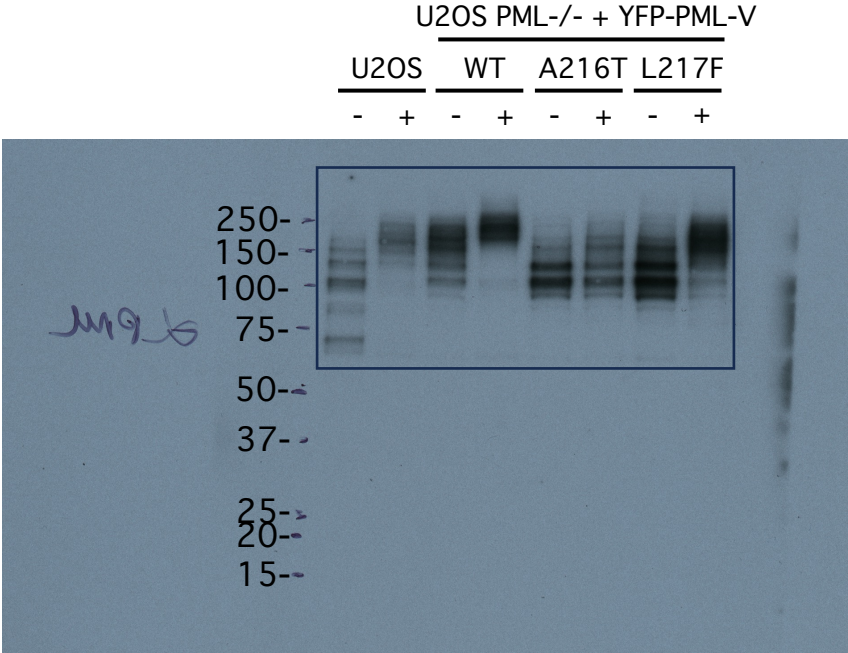

Anti-PML Immuno-Blot

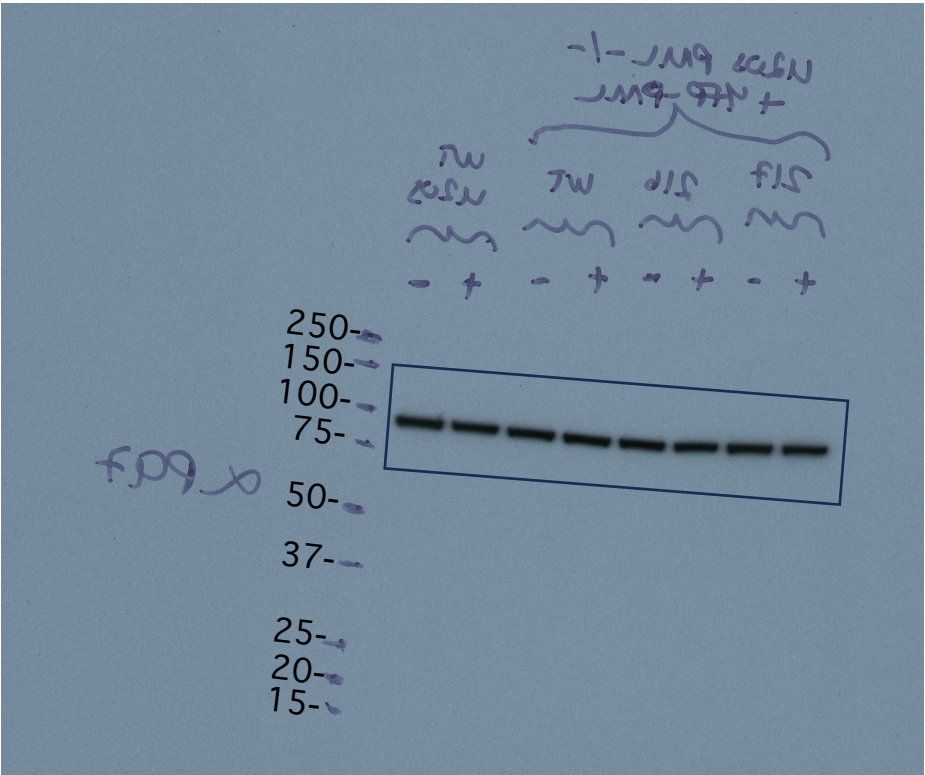

Anti-p97 Immuno-Blot

Supplement: SourceData F5 — is the source file for Fig. 5. [file jcb_202407133_sourcedataf5.pdf]

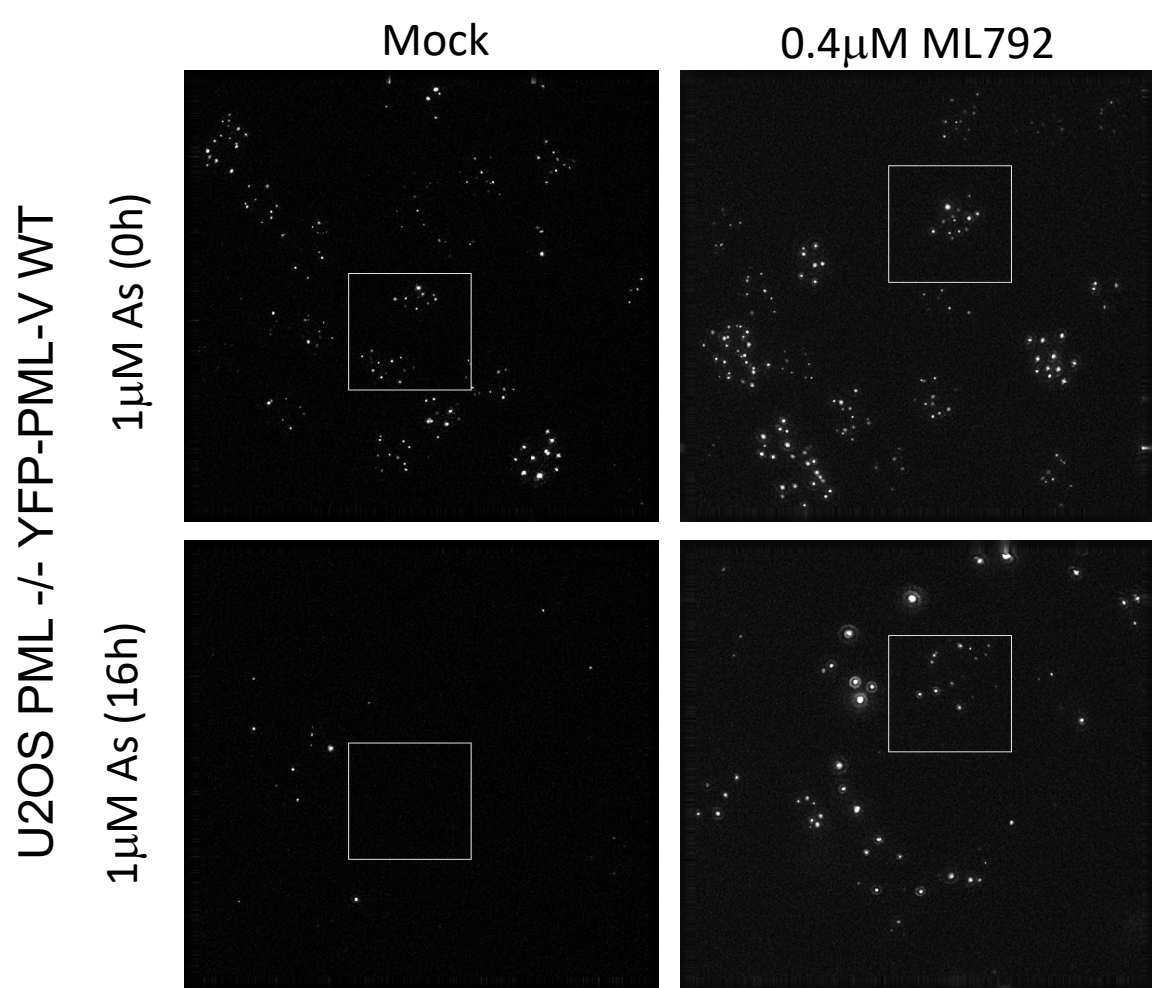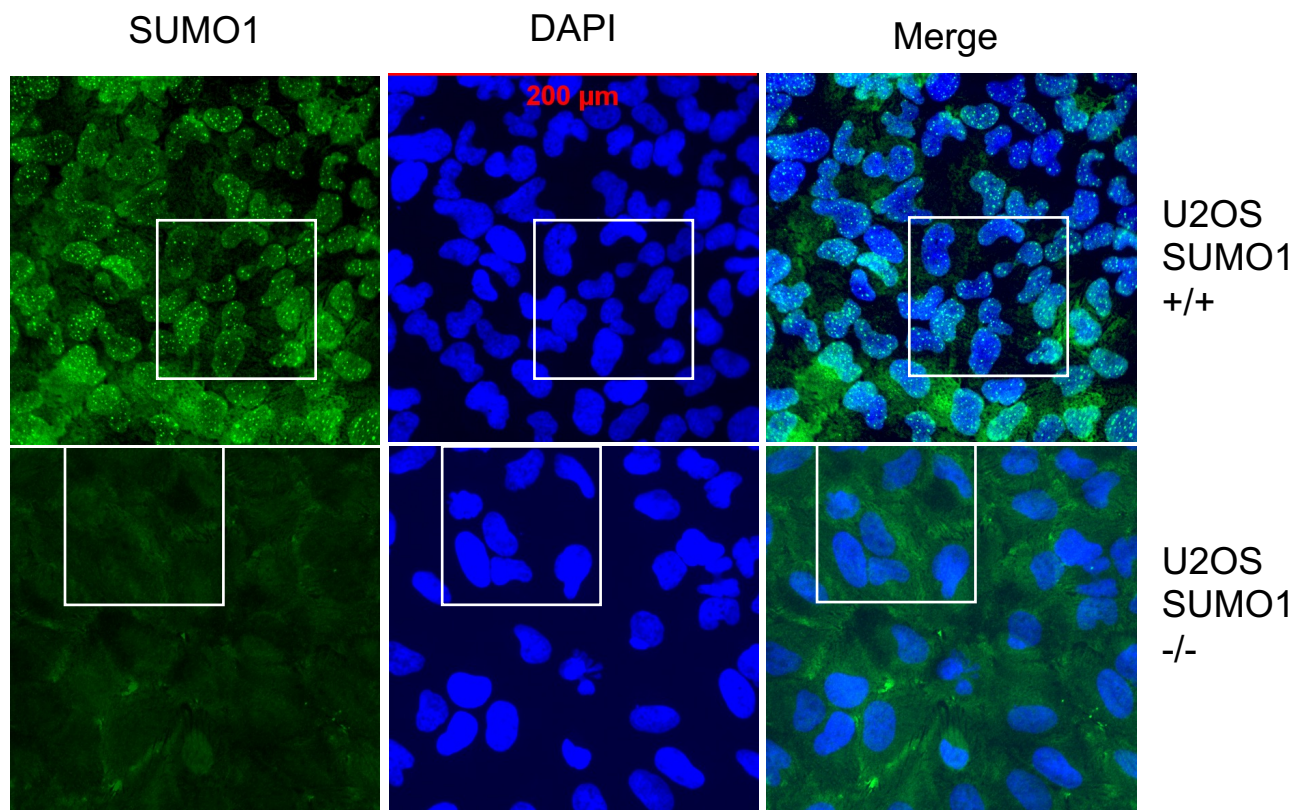

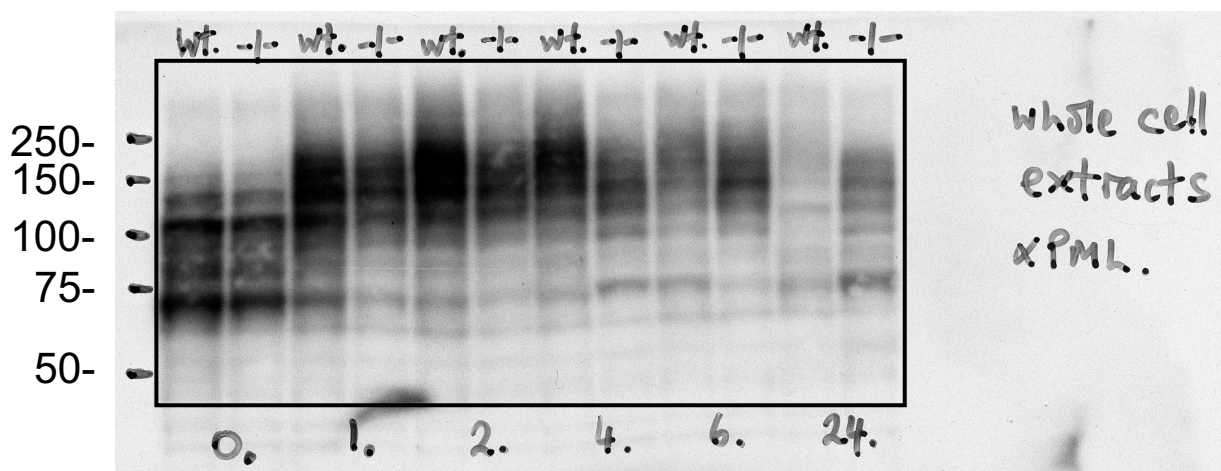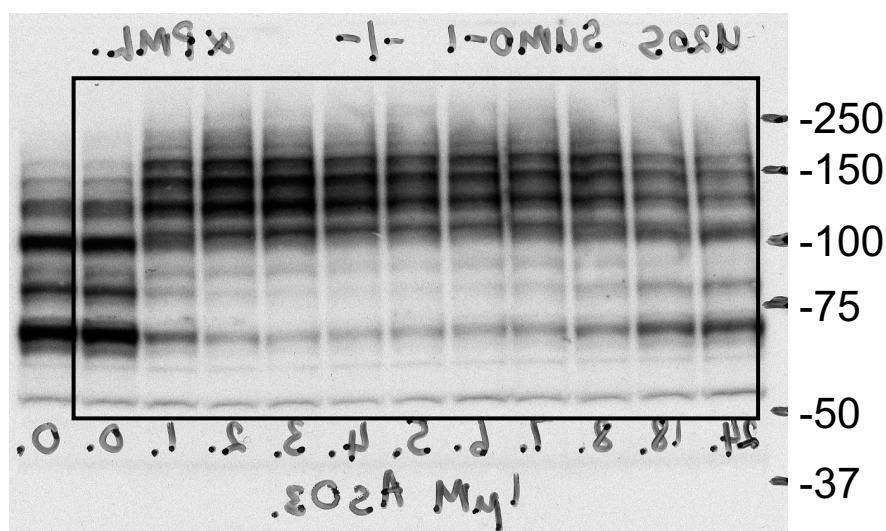

Supplement: SourceData F8 — is the source file for Fig. 8. [file jcb_202407133_sourcedataf8.pdf]

1  $\mu$ M As 2h      -As      +As

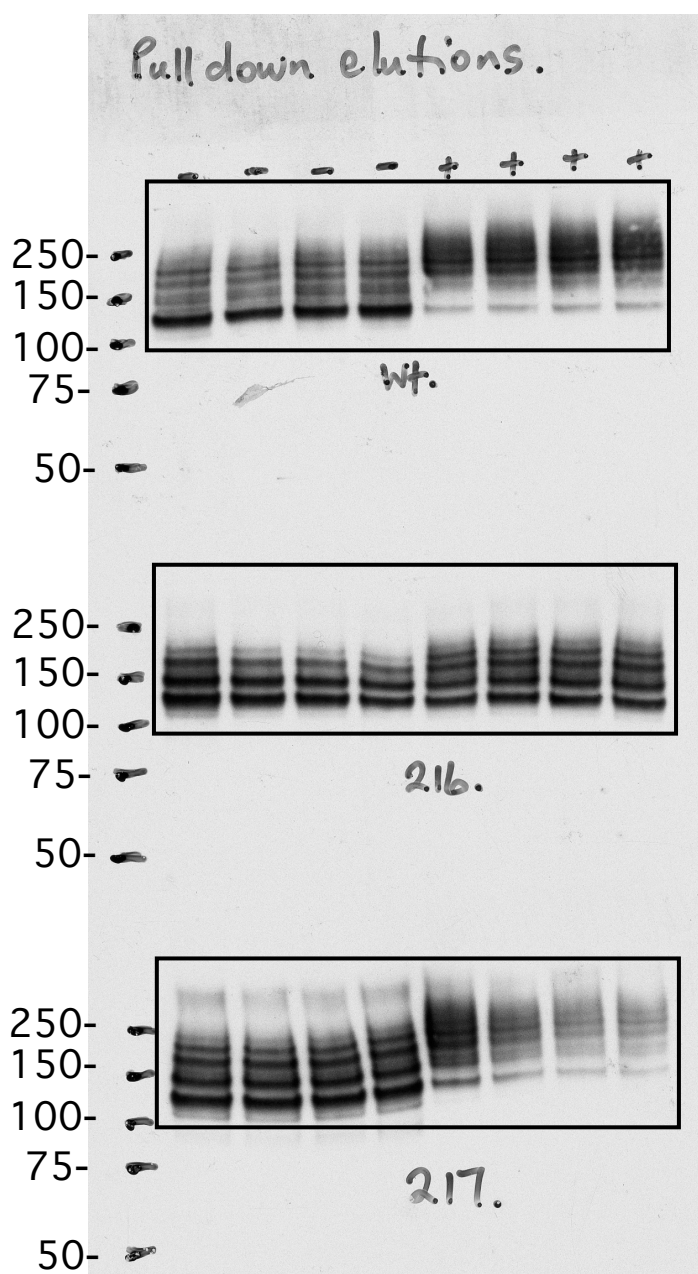

Anti-PML Immuno-Blot

Supplement: SourceData FS1 — is the source file for Fig. S1. [file jcb_202407133_sourcedatafs1.pdf]

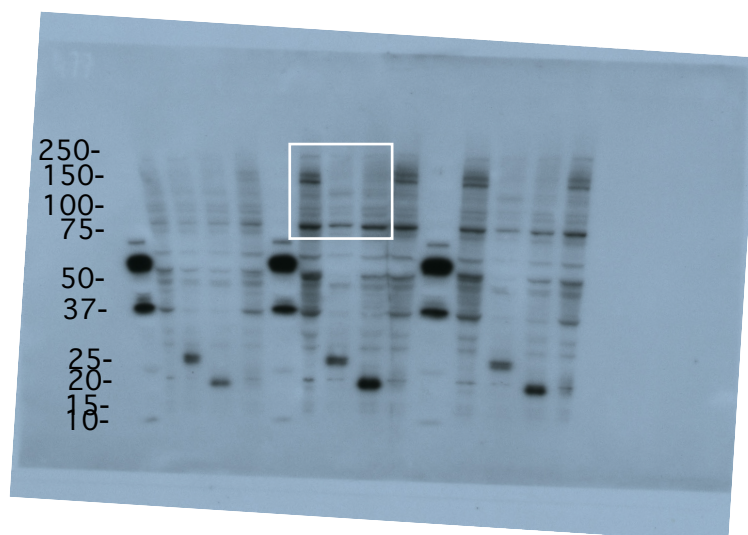

Anti-TOPOPRS  
(mouse)

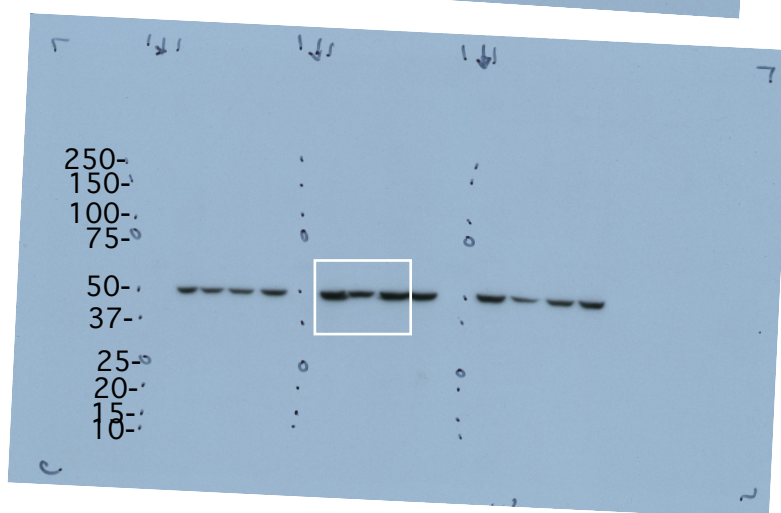

Anti-Tubulin

Cas9 only

Cas9+  
TOPORS#1

Cas9+  
TOPORS#2

1 $\mu$ M As  
(0h)

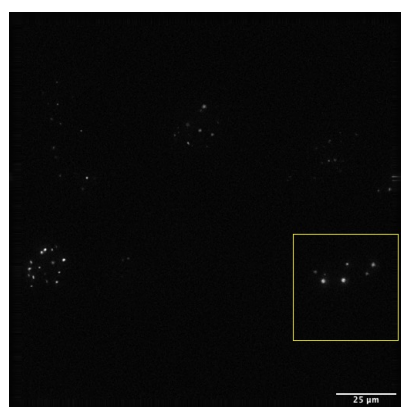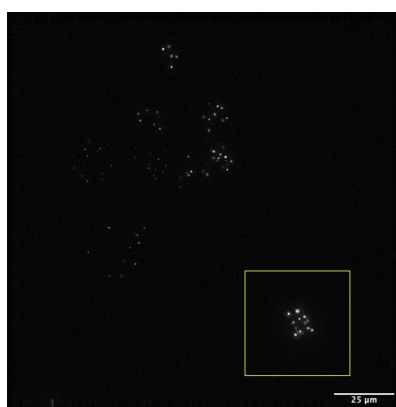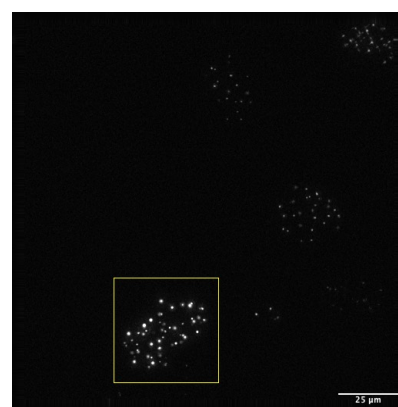

1 $\mu$ M As  
(16h)

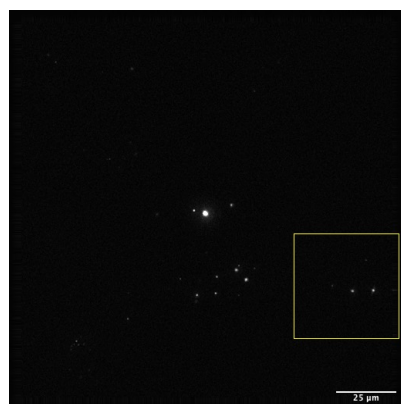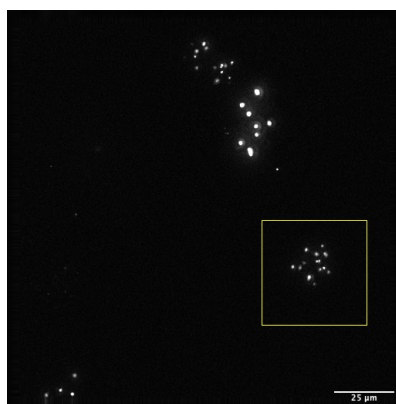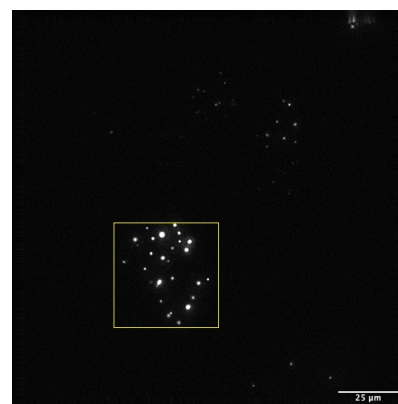

Supplement: SourceData FS7 — is the source file for Fig. S7. [file jcb_202407133_sourcedatafs7.pdf]
