# Supplementary material for: PML mutants from arsenic-resistant patients reveal SUMO1-TOPORS and SUMO2/3-RNF4 degradation pathways
Source: J Cell Biol. 2025 Apr 16;224(6):e202407133. doi: 10.1083/jcb.202407133 (PMC12002637; doi:10.1083/jcb.202407133)
Supplement: SourceData F2 — is the source file for Fig. 2. [file jcb_202407133_sourcedataf2.pdf]

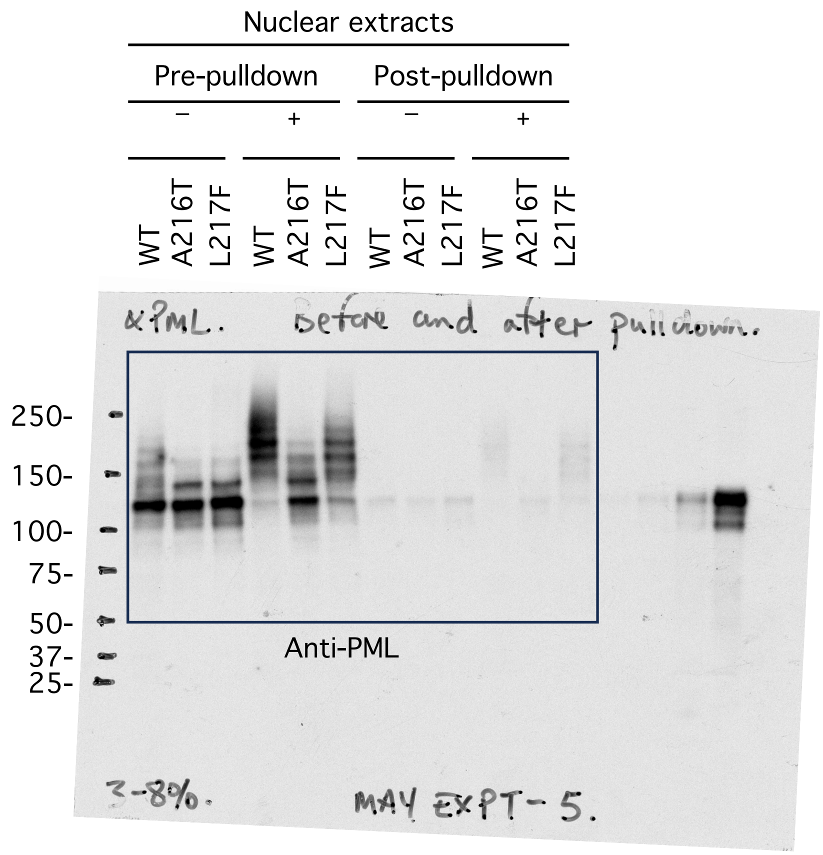

Source Data F2C Jaffray et al 2024

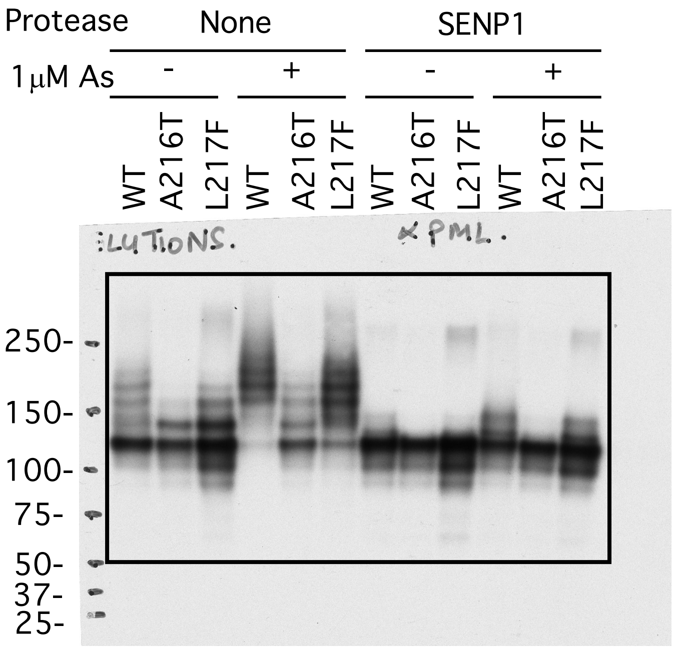

Anti-PML Immunoblot

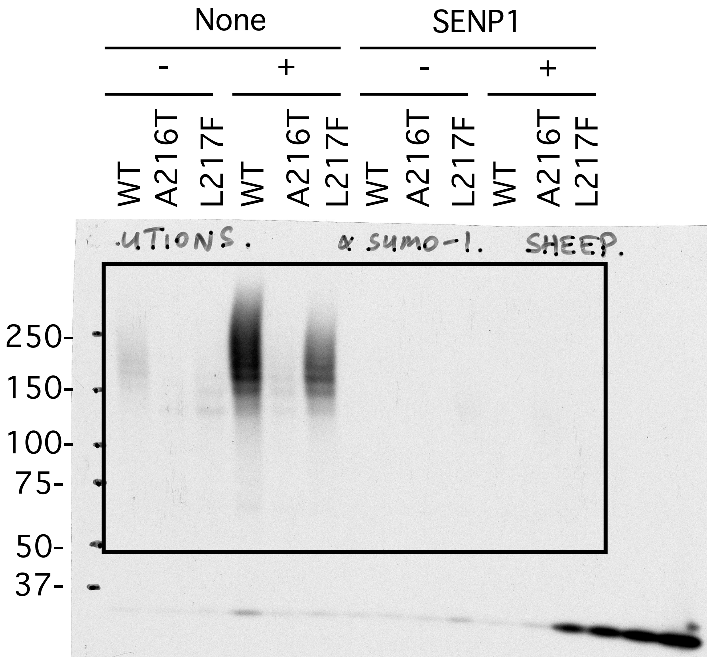

Anti-SUMO1 Immunoblot

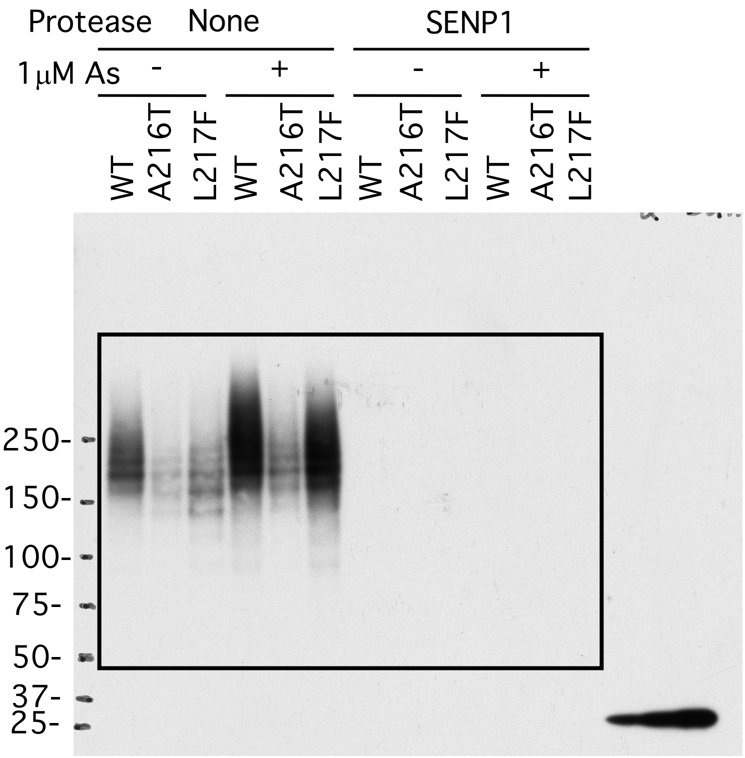

Anti-SUMO2/3 Immunoblot

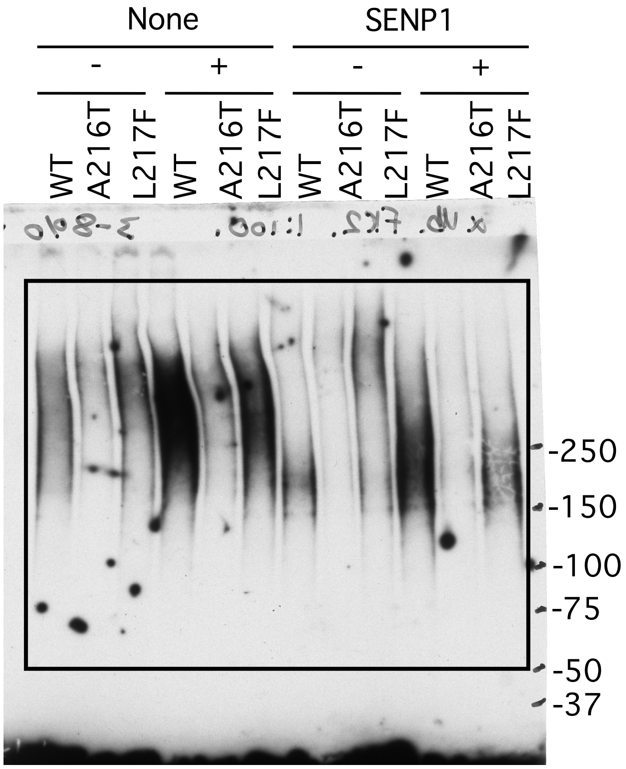

Anti-Ubiquitin Immunoblot

Source Data F2D pt1 Jaffray et al 2024

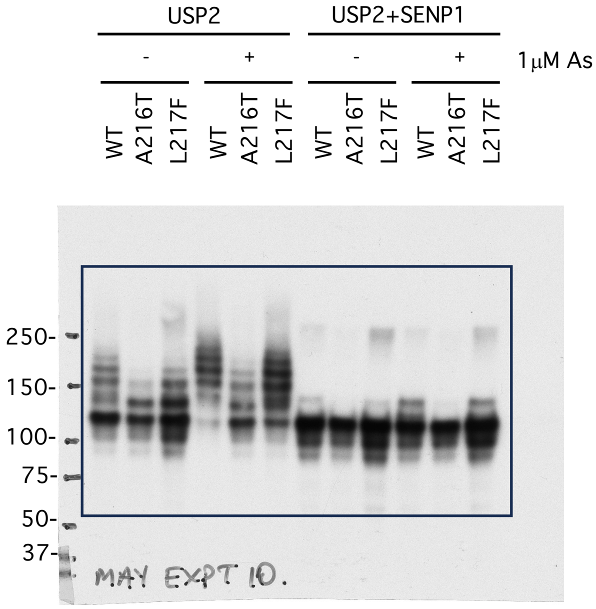

Anti-PML Immuno-Blot

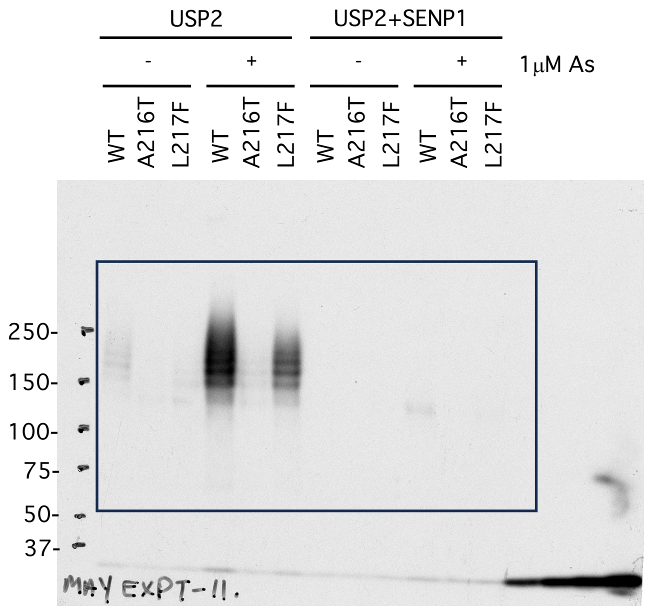

Anti-SUMO1 Immuno-Blot

From Fig 2D pt1 →

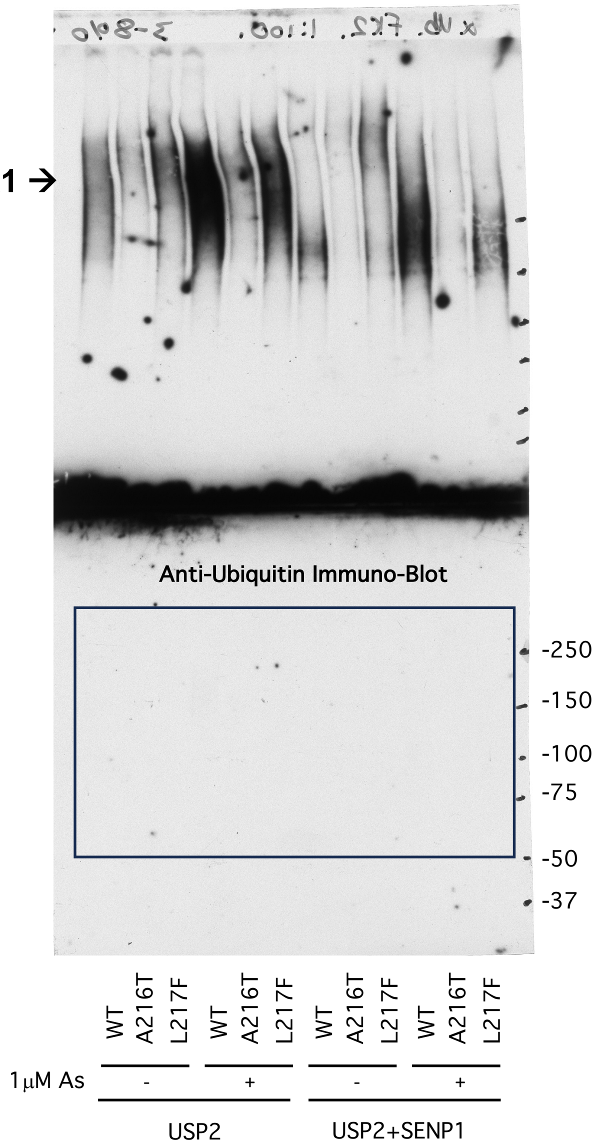

Anti-Ubiquitin Immuno-Blot

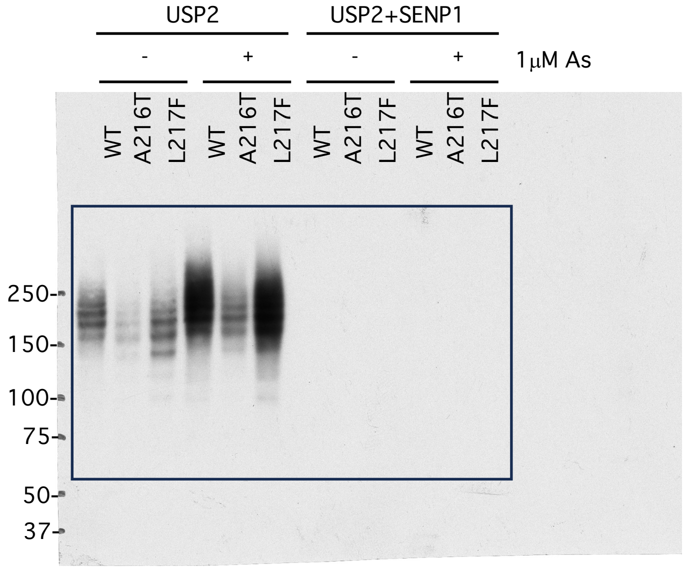

Anti-SUMO2/3 Immuno-Blot

Source Data F2D pt2 Jaffray et al 2024

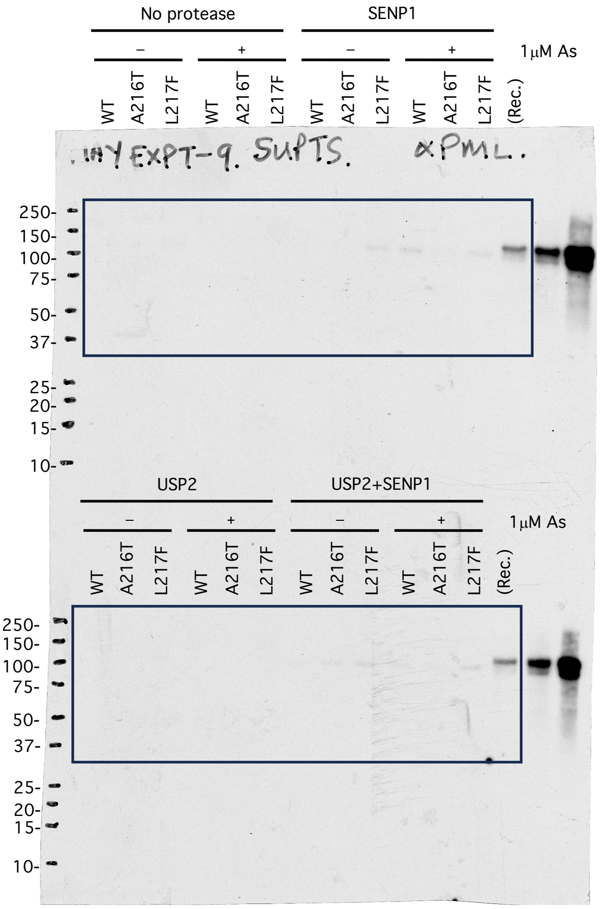

Anti-PML Immuno-Blot

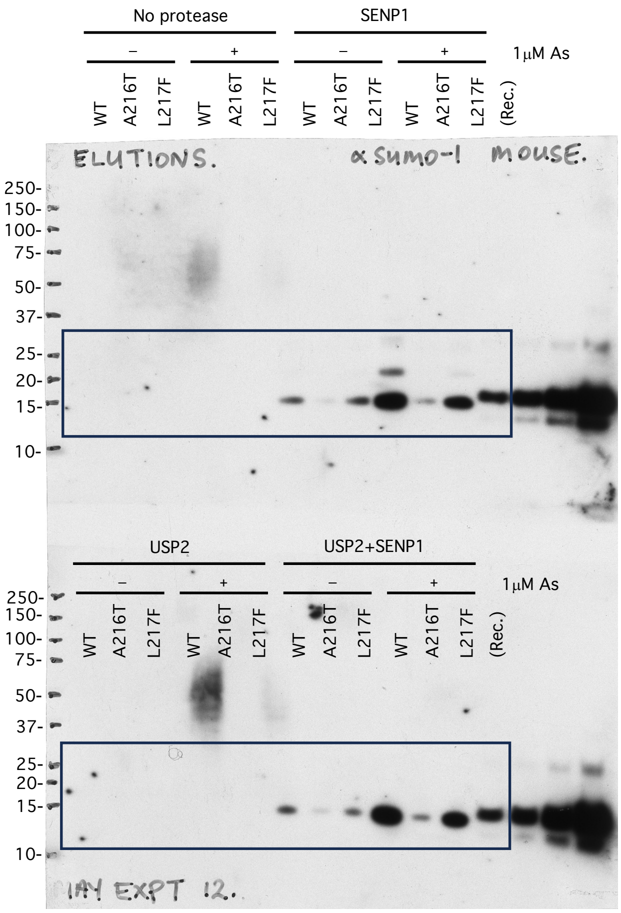

Anti-SUMO1 Immuno-Blot

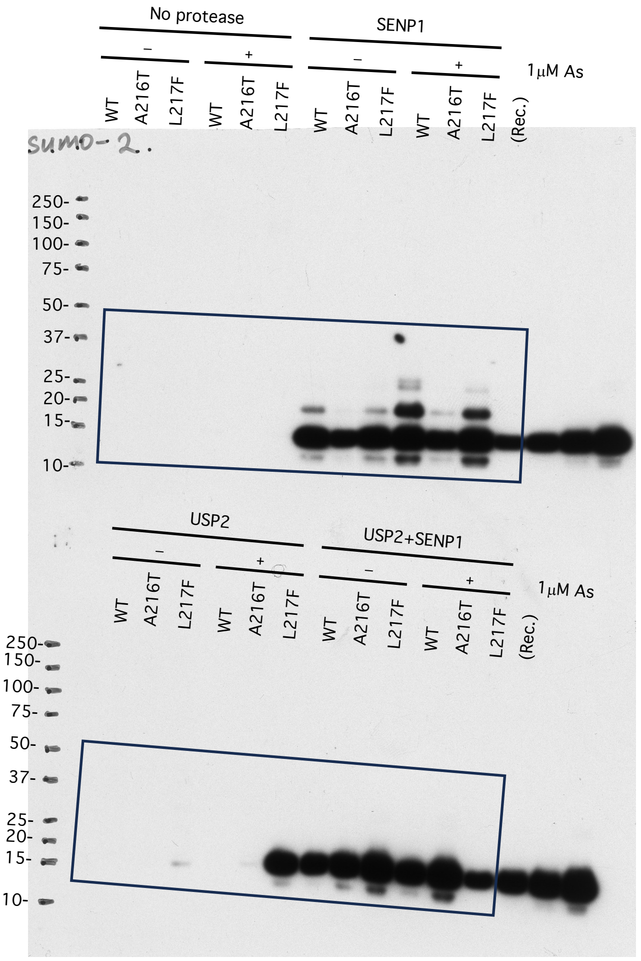

Anti-SUMO2/3 Immuno-Blot

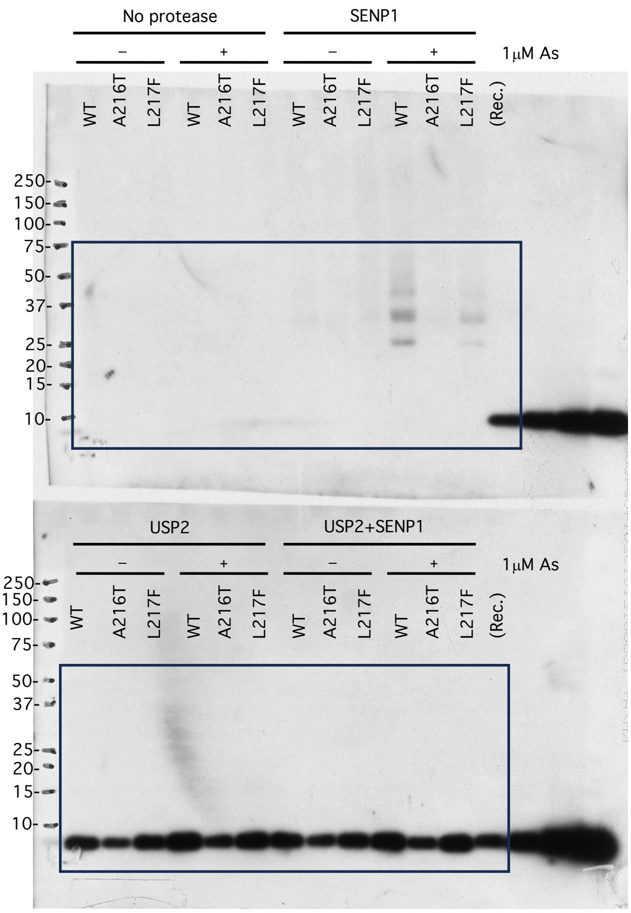

Anti-Ubiquitin1 Immuno-Blot

Source Data F2E Jaffray et al 2024
