# Supplementary material for: PML mutants from arsenic-resistant patients reveal SUMO1-TOPORS and SUMO2/3-RNF4 degradation pathways
Source: J Cell Biol. 2025 Apr 16;224(6):e202407133. doi: 10.1083/jcb.202407133 (PMC12002637; doi:10.1083/jcb.202407133)
Supplement: SourceData F9 — is the source file for Fig. 9. [file jcb_202407133_sourcedataf9.pdf]

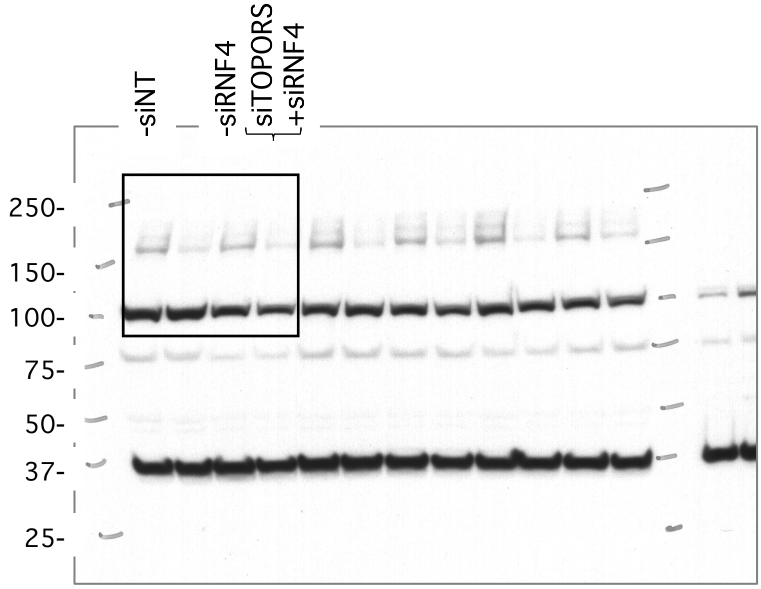

Anti-TOPORS immuno-blot

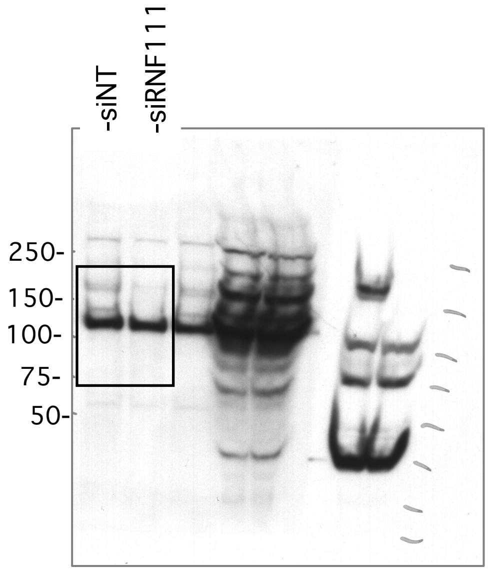

Anti-RNF111 immuno-blot

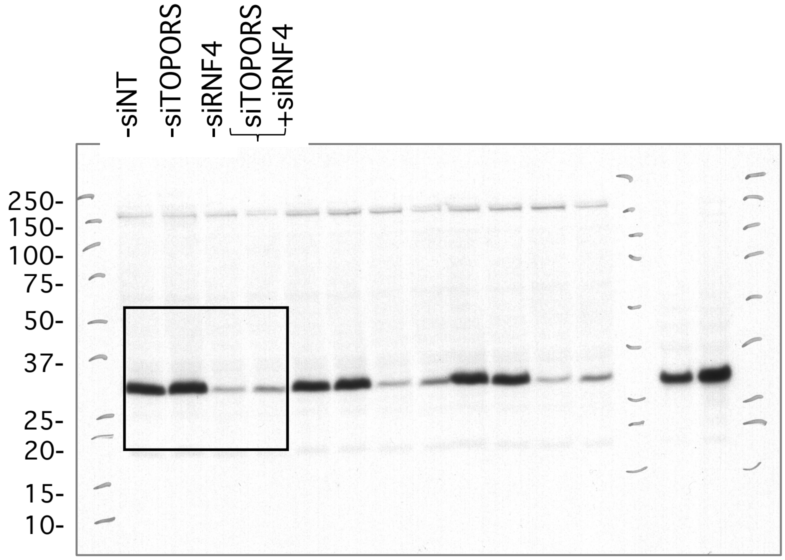

Anti-RNF4 immuno-blot

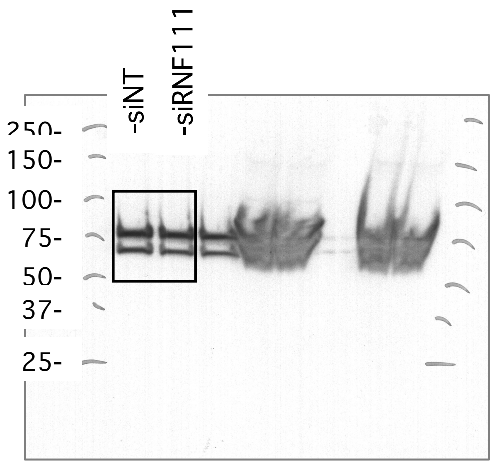

Anti-Lamin A/C immuno-blot

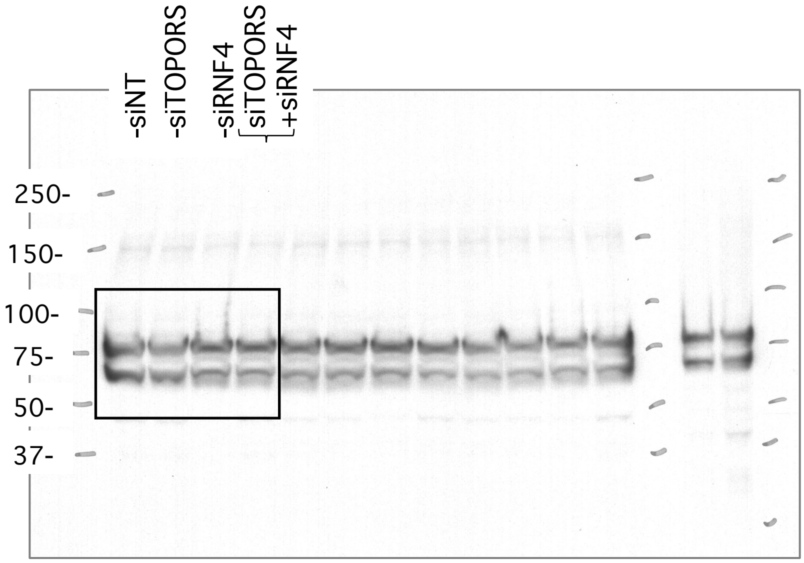

Anti-Lamin A/C immuno-blot

Source Data F9C Jaffray et al 2024

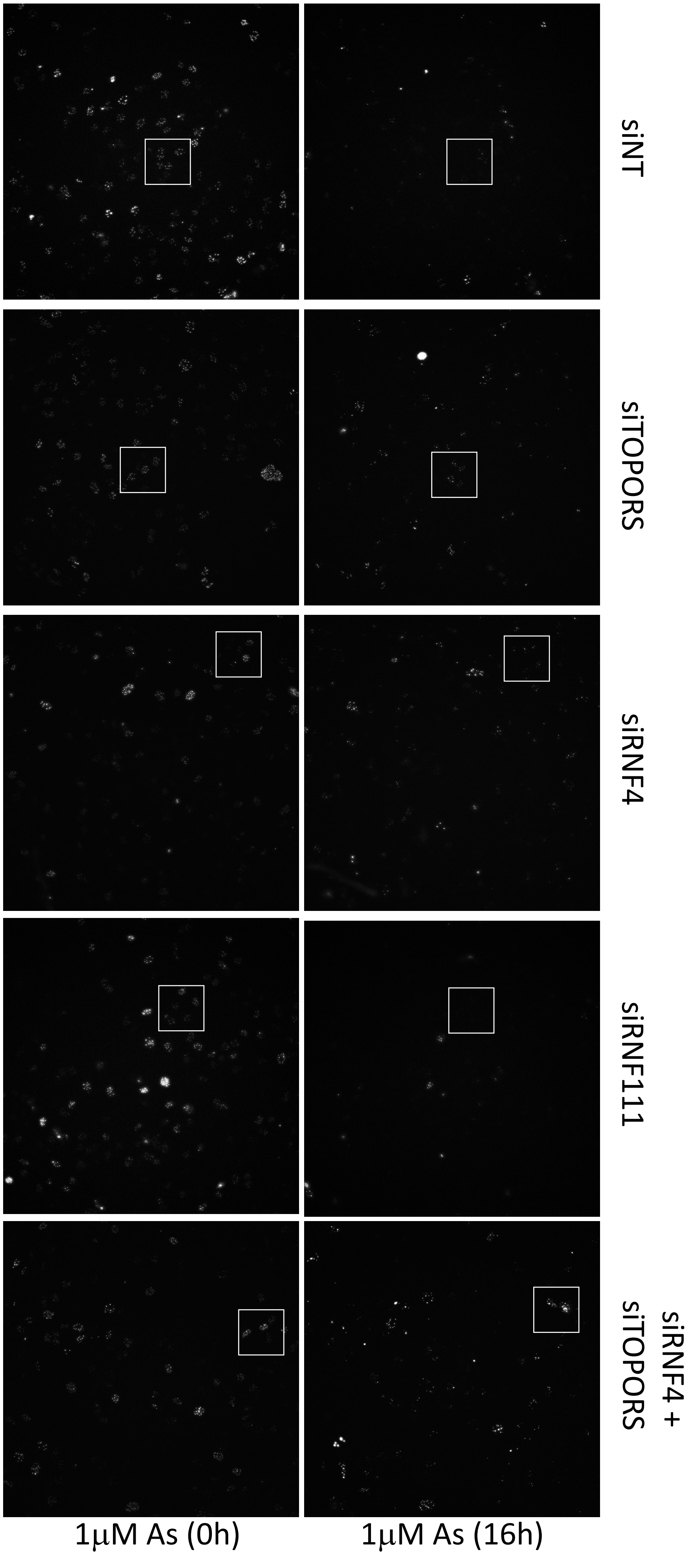

Source Data F9F pt1 Jaffray et al 2024
